# Pattern of respiratory diseases seen among adults in an emergency room in a resource-poor nation health facility

**DOI:** 10.4314/pamj.v9i1.71199

**Published:** 2011-06-30

**Authors:** Olufemi Olumuyiwa Desalu, Ololade Olusola Ojo, Olusegun Adesola Busari, Abayomi Fadeyi

**Affiliations:** 1 Department of Medicine, University of Ilorin Teaching Hospital Ilorin, Nigeria; 2 Department of Community Medicine, Federal Medical Centre Ido-Ekiti Nigeria; 3 Department of Medicine, Federal Medical Centre Ido-Ekiti Nigeria; 4 Department of Medical Microbiology University of Ilorin Teaching Hospital, Ilorin, Nigeria

**Keywords:** Disease pattern, Respiratory Disease, Resource-poor country, Emergency Room, Nigeria

## Abstract

**Introduction:**

There is a paucity of information on utilisation of emergency medical services in Nigeria. This study was conducted to determine the pattern of respiratory diseases seen among adults in an emergency room(ER) and their mortality within twenty- four hours in a health facility in Nigeria.

**Methods:**

We carried out a retrospective study on adult patients that presented with respiratory condition from November 2004 to December 2010 at the emergency room of Federal Medical Centre Ido-Ekiti, south western, Nigeria.

**Results:**

A total of 3671 cases were seen, 368 were respiratory cases accounting for 10.2 % of the total emergency room visitations. The male to female patients ratio was 1.2:1 and their mean was 49 9 ± 20.3 years. Pneumonia (34.5%) was the most common cases seen in the ER, followed PTB (29.4%), acute asthma (24.5%) , acute exacerbation of COPD (10.3%), upper airway tract obstruction and malignant pleural effusion were 0.5% respectively. Fourteen of the PTB cases (3.8%) were complicated by cor-pulmonale, 9(2.5%) by pleural effusion, 4(1.1%) by massive haemoptysis and 2(0.5%) by pneumothorax. Twenty-four hours mortality was 7.4% and 44.4% of the death was due to PTB, 37.0% was due to pneumonia and 14.8% due to acute asthma attack. The overall mortalities also had a bimodal age group distribution as the highest death was recorded in ages 30-39 and ≥70 years.

**Conclusion:**

Pneumonia and PTB were the leading respiratory diseases among adults causing of emergency room visit and early mortality in this health facility in Nigeria.

## Background

Respiratory diseases include a broad range of disease such as acute respiratory infections, pneumonia, obstructive lung diseases, pleural disease and pneumoconiosis as well as respiratory cancers [1]. It imposes a severe burden on the populace and is the major cause of morbidity and mortality worldwide, as 17.4% of all deaths and 13.3% of all Disability-Adjusted Life Years (DALYs) in year 2000 was attributed to five top respiratory diseases [2]. In Nigeria, lower respiratory tract infection was the second leading cause of death, all ages in 2002 [3]. Respiratory diseases were third the leading cause of hospitalisation in Canada and fourth leading cause of disability in the United State of America USA [4, 5]. Individuals with respiratory disease can present to the emergency unit or room as acute illness or as exacerbation of chronic respiratory disease. In the United Kingdom(UK), respiratory disease is the second most common illness responsible for emergency admission to hospital and cost the UK government £6.6 billion in 2004 [6]. In most developing countries and resource poor nation adequate provisions of good medical are not widely available and the burdens of respiratory diseases are not well known. Some studies have been carried out in Nigeria and other African on pattern of medical cases admitted as an emergency, but none on the pattern of respiratory diseases presenting as an emergency among adult patients in sub-Saharan Africa [7–9]. This study was conducted to determine the pattern of respiratory diseases seen among adults in an emergency room (ER) and their mortality within twenty- four hours in a health facility in Nigeria.

## Methods

This retrospective study was carried out at the Federal Medical Centre Ido-Ekiti in south western, Nigeria. The study was approved by the research and ethical committee of the hospital. The study center is a 250-beds tertiary hospital that serves as a referral hospital for Ekiti and its adjoining states, the hospital also run an internship and postgraduate training for interns and resident doctors respectively.

The hospital is located in a tropical climate with two seasons: a wet (rainy) season from April to October and a dry season from November to March, the wettest month was June. The average annual rainfall was 1,770 mm (70 inches). Average temperature ranges are from 23 degrees Celsius (73 degrees Fahrenheit) to 32 degrees Celsius (90 degrees Fahrenheit) all year [10].

The study used a retrospective design. We retrieved medical records of patients (aged ≥18years) with respiratory disease that visited the emergency unit of the hospital from November 2004 to December 2010 from the Health Record Department of the hospital. The medical record files were reviewed and the socio-demographic information, presenting complaints, seasons of admission, outcome of management of patients were extracted and entered into the data sheet by designated registrars in the department of medicine.

The patients with missing medical record files, uncompleted vital clinical information and whose diagnosis did meet the respiratory diseases guidelines [11–15] were excluded from the study. The data collected was analysed using statistical package for social sciences (SPSS version 15). Univariate analysis was performed and descriptive and frequency statistics were obtained for the obtained variables. Pearson Chi square test was used to determine the significance of categorical variables and P value of < 0.05 was considered significant. Spearman correlation coefficient was used to determine the association between 24 hour mortality and socio-demographic variables.

## Results

A total of 3671 adult cases were seen in the emergency room from November 2004 to December 2010. Of the 3671 cases, 368 were respiratory diseases accounting for 10.2% of the total emergency room visitation in the hospital (Table 1). The mean age of the patients was 49 9 ± 20.3 years. One hundred and ninety nine (54.1%) were males and 169 (45.1%) were females with a male to female ratio of 1.2:1. The age and sex distributions showed that 80 (21.7%) of the patients were in age group ≥ 70 years. Nearly one third of the patients affected were aged ≥ 60 years (Table 2).

**Table 1:** Systemic distribution of cases seen in the emergency room of the Federal Medical Centre Ido-Ekiti, south western, Nigeria, from November 2004 to December 2010 (N=3671)

Diagnosis	N	(%)
Surgery	1150	31.3
Respiratory	368	10.2
Gastroenterology	366	10.0
Cardiology	334	9.2
Neurology	223	6.1
Endocrinology	168	4.6
Heamatology	98	2.7
Psychiatry	61	1.7
Renal	45	1.2
O& G	175	4.8
Others	629	17.1

Others: Sepsis, malaria, HIV/AIDS, snake bite

**Table 2:** Age and Sex distribution of patients with respiratory diseases seen in the emergency room of the Federal Medical Centre Ido-Ekiti, south western, Nigeria, from November 2004 to December 2010

Age range	Female (N)	Male (N)	Total
18-19	11	5	16
20-29	27	26	53
30-39	36	27	63
40-49	11	32	43
50-59	29	27	56
60-69	24	33	57
70+	31	49	80
**Total**	**169**	**199**	**368**

Out of the 368, 127(34.5%) had pneumonia, 108(29.4%) had complicated and uncomplicated PTB, 90(24.5%) had acute asthma attack while 38(10.3%) had acute exacerbation of COPD. Further analysis of those with PTB revealed that 79(21.5%) had uncomplicated PTB, 14(3.8%) had associated cor-pulmonale, 9(2.5%) were associated with pleural effusion, 4(1.1%) were associated with massive haemoptysis and 2(0.5%) had associated pneumothorax (Table 3). The mean ages, sex ratios and case specific mortalities within 24 hours after ER visitation of the four leading respiratory conditions are shown in Table 4. More than half of the patients with respiratory condition (56.5%) were seen in the ER during the wet (rainy) season and 160 (43.5%) during the dry season.

**Table 3:** Respiratory diseases seen at the emergency room of the Federal Medical Centre Ido-Ekiti, south western, Nigeria, from November 2004 to December 2010

Respiratory Diseases	N (%)
Pneumonia	127 (34.5)
Pulmonary tuberculosis (All)	108 (29.4)
-Uncomplicated	79 (21.5)
+Cor- pulmonale	14 (3.8)
+Pleural effusion	9 (2.5)
+Massive haemoptysis	4 (1.1)
+Pneumothorax	2 (0.5)
Acute asthma	90 (24.5)
Acute exacerbation of COPD	38 (10.3)
Upper airway obstruction	2 (0.5)
Malignant Pleural effusion	2 (0.5)
Acute chest syndrome	1 (0.3)

**Table 4:** Analysis of some variables associated with respiratory diseases seen in the emergency room of the Federal Medical Centre Ido-Ekiti, south western, Nigeria, from November 2004 to December 2010

Respiratory diseases	Mean age(yrs)	Sex ratio (M:F)	Case specific mortality in 24 hours (%)
Pneumonia	50.4(22.0)	1:1.5	7.1
Pulmonary tuberculosis	44.8(19.8)	1.1:1	7.4
Asthma	47.5(18.4)	1:1.4	4.4
Chronic Obstructive Pulmonary Diseases (COPD)	63.8(13.5)	2.5:1	2.6

**Figure 1:**
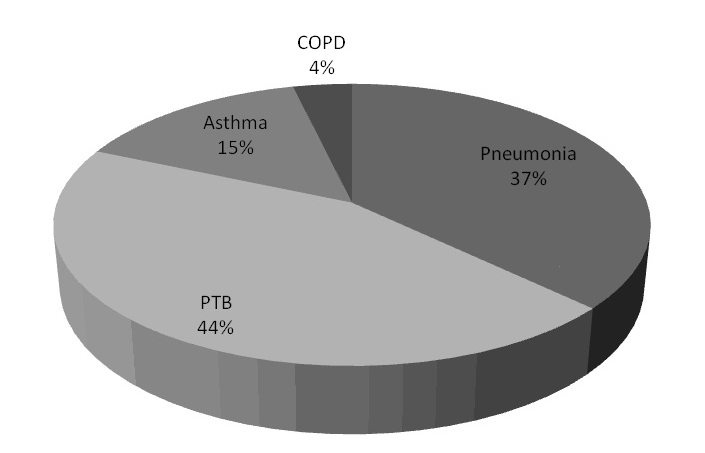
Mortality distribution according to respiratory diseases in the emergency room of the Federal Medical Centre Ido-Ekiti, south western, Nigeria, from November 2004 to December 2010

A total 27(7.4%) patients died within 24hours of admission in the emergency room. This result also showed that 44.4% of the total mortality was due to PTB, pneumonia (37.0%), acute asthma (14.8%) and COPD (3.7%) (Figure 1). Furthermore ages 30-39 and ≥ 70years had the highest mortality rate. There was no statistically significant differences in death rates in both sexes (51.9% vs.48.1% p=0.352). Spearman correlation coefficient showed no correlation between 24 hour mortality and socio-demographic variables: age (r^2^ = +0.02, p=0.64), sex (r^2^ =-0.01, p= 0.98) and socioeconomic status (r^2^ = + 0.04, p= 0.41).

## Discussion

Our study has attempted to determine the pattern of respiratory disease causing emergency room visit among adults? patients in a health facility in Nigeria and afforded us the opportunity to have insight into the burden of respiratory diseases in emergency room in resource poor countries. This present study revealed that respiratory diseases accounted for approximately 10 % of the total hospital emergency room visit. This result is higher than 5.9% in the city of Gama, Brazil but closer and comparable to 13% in the United Kingdom, however it is less than 16.2% in Jamaica, West Indices [4,16,17].

We found that pneumonia which is a lower respiratory tract infection accounted for 34.5% of the cases, and was the leading cause of visit to the emergency room in our centre. This was followed by PTB (29.4%), acute asthma attack (24.5%) and acute exacerbation of COPD (10.3%). Further analysis of the subset of PTB patients showed that 14(13%) was complicated by cor pulmonale and 9(8.3%) by pleural effusion.

This result is similar to a study in Al-Kharj, Kingdom of Saudi Arabia were respiratory tract infection was found to be the commonest respiratory disease causing emergency room visit [18]. This study is in contrast to findings in Brazil and United Kingdom (UK) where upper respiratory tract infection and chronic obstructive pulmonary disease (COPD) are the commonest causes of ER visitation respectively [4].

The geographical variation in ranking of utilisation of emergency service by patients with respiratory disease may be due to differences in the predisposing risk factor for respiratory diseases like population ageing, urbanization, tobacco smoking, HIV/AIDS and environmental pollution.

In this study, nearly one third of the patients seen were ≥60 years of age, which is similar to the result in UK [4]. This trend can be attributed to age related co- morbid medical conditions like heart failure, chronic obstructive pulmonary disease and Diabetes mellitus that predispose patients to respiratory illness [2]. Moreover aging has been associated with depression of body immunity and is known to enhance susceptibility to infection [2]. The elderly subjects as compared with young and middle aged population are also highly susceptible to acute lower respiratory infection [4,9]. Dominici and colleagues in the year 2006 reported that short-term exposure to fine particle air pollution are dangerous, and significantly increases the risk for cardiovascular and respiratory disease among people over the age of 65 years [19].

This study showed that more patients with respiratory illness were seen in the emergency room in the wet (rainy) season, (56.5% vs. 43.5%) but result was not statistically significant. The slight increase in the number of cases seen during the wet season may be due to seasonal increase in the level of humidity, airborne allergen such as pollens and increased indoor air pollution from the use of solid fuel for cooking and poor ventilation from closed window. Some conditions like asthma and PTB have been documented to have an increase in the rate of hospitalization during rainy season while pneumonia is reported to be more common in the dry season [20,21].

Mortality within 24 hours of admission into the emergency room was 7.4%. Approximately 40% of the total mortality was due to PTB and pneumonia and these two conditions are the leading causes of early mortality among adult patients with respiratory diseases seen in the ER in our facility. Our result is in agreement with a study in south western Nigeria [22]. The mortalities also had a bimodal age group distribution as the highest death was recorded in ages 30-39 and ≥70 years. The socio-demographic variables were not significantly associated the mortality.

Despite the limitation of retrospective study which is often characterized by poor and incomplete medical records keeping and follow up visitation, we have tried to establish the pattern of respiratory diseases seen in the emergency room visit in this study.

## Conclusion

Pneumonia and PTB were the leading causes of adult respiratory diseases seen in the emergency room in this health facility in Nigeria. In most resource poor countries of which Nigeria is inclusive, inadequate funds are budgeted for health care. Therefore it is imperative to give more priority in terms of allocation of resources to the leading respiratory diseases causing emergency room visit.
